# Standardized uptake value_max_ of the primary lesion combined with tumor markers for clinically predicting distant metastasis in de novo lung adenocarcinoma

**DOI:** 10.1002/cam4.6961

**Published:** 2024-03-29

**Authors:** Baoli Jin, Xiaolian Wen, Hanji Tian, Hongxia Guo, Mingyan Hao, Jing Wu, Xiaomin Li, Yuejun Ren, Xin Wang, Xiaolu Ren

**Affiliations:** ^1^ Department of Radiation Oncology, Shanxi Province Cancer Hospital, Shanxi Hospital Affiliated to Cancer Hospital Chinese Academy of Medical Sciences, Cancer Hospital Affiliated to Shanxi Medical University Taiyuan China; ^2^ Department of Oncology, Shanxi Province Cancer Hospital, Shanxi Hospital Affiliated to Cancer Hospital Chinese Academy of Medical Sciences, Cancer Hospital Affiliated to Shanxi Medical University Taiyuan China; ^3^ Department of Surgery, Shanxi Province Cancer Hospital, Shanxi Hospital Affiliated to Cancer Hospital Chinese Academy of Medical Sciences, Cancer Hospital Affiliated to Shanxi Medical University Taiyuan China; ^4^ Shanxi Bethune Hospital Taiyuan Shanxi China; ^5^ Department of Administration, Shanxi Province Cancer Hospital, Shanxi Hospital Affiliated to Cancer Hospital Chinese Academy of Medical Sciences, Cancer Hospital Affiliated to Shanxi Medical University Taiyuan China; ^6^ Department of MR/CT, Shanxi Province Cancer Hospital, Shanxi Hospital Affiliated to Cancer Hospital Chinese Academy of Medical Sciences, Cancer Hospital Affiliated to Shanxi Medical University Taiyuan China; ^7^ Department of Surgery First Hospital of Shanxi Medical University Taiyuan China

**Keywords:** distant metastasis, lung adenocarcinoma, prediction, retrospective observational study, tumor marker

## Abstract

**Background:**

To examine standardized uptake value_max_ of the primary lesion (pSUVmax) and tumor markers (TMs) for clinically predicting distant metastasis in novo lung adenocarcinoma.

**Methods:**

The current retrospective observational study examined individuals diagnosed with de novo lung adenocarcinoma at Shanxi Cancer Hospital between February 2015 and December 2019.

**Results:**

Totally, 532 de novo lung adenocarcinoma cases were included. They were aged 60.8 ± 9.7 years and comprised 224 women and 268 patients with distant metastasis. The areas under the curves (AUCs) of pSUVmax, lactate dehydrogenase (LDH), carcinoembryonic antigen (CEA), cytokeratin‐19 fragment (CYFRA21‐1), carbohydrate antigen 125 (CA125), and Grade of TMs for predicting distant metastasis were 0.742, 0.601, 0.671, 0.700, 0.736, and 0.745, respectively. The combination of pSUVmax, LDH, CEA, CYFRA21‐1, CA125, and grade of TMs in predicting distant metastasis had an AUC value of 0.816 (95%CI: 0.781–0.851), with sensitivity of 89.2%, specificity of 58.7%, positive predictive value of 73.7%, and negative predictive value of 79.7%, respectively.

**Conclusions:**

pSUVmax combined with serum levels of LDH, CEA, CYFRA21‐1, CA125, and the grade of TMs may have good performance in predicting distant metastasis of de novo lung adenocarcinoma.

## 1 INTRODUCTION

Lung cancer represents one of the commonest malignancy and contributes to 13% of newly diagnosed carcinomas.^1^, ^2^, ^3^, ^4^ According to histological type, non‐small cell lung cancer (NSCLC) comprises >80% of all lung cancer cases, with the commonest subtype being adenocarcinoma.^5^, ^6^ Most newly diagnosed lung cancers are metastatic at presentation, especially in adenocarcinoma which are prone to distant metastasis.^7^, ^8^ Due to the elevated odds of metastasis, lung cancer is characterized by ineffective therapies and poor prognosis.^9^ The 5‐year overall survival of lung cancer following diagnosis is <20%, for a 5‐year relative survival of only 5% in lung adenocarcinoma.^10^, ^11^ Therefore, the diagnosis of metastasis is crucial for individualized systemic therapeutic strategies and prognosis evaluation in lung cancer, especially the evaluation of distant metastasis of lung adenocarcinoma, which requires more attention in clinical practice.

Pathological examination is the gold standard to diagnose metastasis, but it will bring certain trauma to patients.^12^ Computed tomography (CT) can provide certain information about metastasis, but cannot detect the lesions <0.4 cm.^10^ Currently, Fluorine‐18 fluorodeoxyglucose positron‐emission tomography/computed tomography (18F‐FDG PET/CT), a newly developed noninvasive imaging device, has been wildly used in the staging of lung cancers. A previous study has typically proven that the standardized uptake value_max_ (SUVmax) of more than 2.5 can distinguished malignant from benign lesions.^13^ However, in the diagnosis of distant metastasis, SUVmax of 2.5 was not an appropriate threshold and the cutoff of SUVmax of the primary lesion (pSUVmax) was still controversial. What's more, in small lesions with diameters less than 1.0 cm, the accuracy of metastatic diagnosis declined drastically, the efficacy was limited when only considering SUVmax of certain lesions.^12^ In addition, the sensitivity and specificity of SUVmax to diagnose brain metastasis were still not satisfactory.^14^, ^15^ For these reasons, a study focused on the diagnostic value of pSUVmax in recent years.^9^ However, in the diagnosis of distant metastasis of de novo lung adenocarcinoma, there are still 15% to 20% false positives and about 20% false negatives in 18F‐FDG PET/CT.^16^, ^17^ Besides the pSUVmax, more information like tumor markers are also needed to improve the accuracy of distant metastatic diagnosis.

Tumor markers (TMs) such as the carcinoembryonic antigen (CEA), neuron‐specific enolase (NSE), and progastrin‐releasing peptide (ProGRP) have important functions in lung cancers. The National Academy of Clinical Biochemistry (NACB) as well as the European Group on Tumor Markers (EGTM) recommended TMs for cancer diagnosis, treatment monitoring, prognostic evaluation, and recurrence assessment.^4^, ^18^ Besides, serum levels of Lactate dehydrogenase (LDH) are significantly increased in stage IV NSCLC and show independent associations with metastatic burden in NSCLC.^14^ However, the above markers are not currently utilized for diagnosing distant metastasis of de novo lung adenocarcinoma.

Therefore, this study aimed to examine the predictive value of pSUVmax combined with serum TMs for the clinical prediction of distant metastasis in de novo lung adenocarcinoma.

## 2 METHODS

### 2.1 Study design and patients

The current retrospective observational study analyzed individuals diagnosed with de novo lung adenocarcinoma at Shanxi Cancer Hospital between February 2015 and December 2019.

Inclusion criteria were as follows: (1) de novo lung adenocarcinoma diagnosed through pathological and immunohistochemical analysis; (2) no previous anti‐tumor therapies; (3) completed ^18^F‐FDG PET/CT and TMs detection for diagnosis of distant metastasis within 1 month; and (4) age ≥ 18 years. Exclusion criteria were as follows: (1) previous cancer diagnosis or multiple pathological types; (2) insufficient laboratory or clinical data; and (3) relevant concurrent disease, such as impaired function of a major organ, hepatobiliary disease, diabetes, moderate‐to‐severe heart disease, moderate‐to‐severe kidney disease, acute pneumonia, or pancreatitis, which may affect influence the results of PET/CT, LDH, and TMs.

The current study had approval from the Ethics Committee of Shanxi Cancer Hospital, who waived the requirement for informed consent because of the retrospective design.

### 2.2 Pathological and immunohistochemical analysis

Tissue samples were obtained by surgical removal or biopsy of primary tumor, primary lymph node, or distant lymph node, and local or distant metastasis. Then, the samples were soaked in 10% neutral formalin and subsequently inserted in paraffin wax. Slices (3 μm) of the samples were taken, and one of them was used for hematoxylin and eosin (H&E) staining, and the remaining were used for TTF‐1 and Napsin A staining. In brief, the tissues were analyzed by the following antibodies: TTF‐1 (clone 8G7G3/1, ZhongShan Jinqiao Biotech., Beijing, China) and Napsin A (multi‐clone, Fuzhou Maixin Biotech Inc., Fuzhou, China). Immunohistochemistry (IHC) was performed on formalin‐fixed paraffin‐embedded (FFPE) lymph node specimens by using a Roche BenchMark®XT autostainer (Roche, Switzerland). At the same time, positive and negative controls were used in each experiment. The results of pathological diagnosis and IHCs were interpreted by at least 2 skilled pathologists with over 5 years of work experience and positive for brown or tan nuclear appearance. TTF‐1 and Napsin A were considered to be expressed with >5% and 50% of tumor cells showing positivity after nuclear staining, respectively.^19^

### 2.3 Data collection and definition

Data on demographic (including age, gender, smoking history), TMs detection (including tumor node metastasis classification (TNM), thyroid transcription factor 1 (TTF‐1), Naspin A and cytokeratin 7 [CK7]), distant metastasis outcomes, and 18F‐FDG PET/CT imaging were retrospectively abstracted from computerized medical records by clinical staff. If any data were missing from the records, this was checked with the doctor or patient to see whether it could be included. If the data were still unavailable, the case was excluded from the study according to the exclusion criteria.

The diagnosis of metastasis was established on the basis of clinical workup procedures, which included CT, ultrasound, magnetic resonance imaging (MRI), positron‐emission tomography (PET) scanning, emission CT, fine‐needle aspiration biopsy, pleural fluid cytology, and/or biopsy. The definition of metastasis and TNM staging followed the International Association for the Study of Lung Cancer (IASLC) eighth TNM classification Lung Cancer Staging System; pathological and metastasis diagnoses were confirmed by ≥2 pathologists and physicians with more than 5 years of experience. If both pathologists were considered to be distant metastasis, it was considered to be distant metastasis. If both pathologists do not match, the case was eliminated from the study.

CEA, CYFRA21‐1, NSE, and CA125 amounts were assessed with specific enzyme‐linked immunosorbent assay (ELISA) manufactured by IDL Biltech (Sweden). LDH amounts were examined with an LDH kit (DiaSys, China). The measurement accuracy of the instrument was determined by the laboratory staff. The laboratory data were based on the results of laboratory examination in our hospital, which was reviewed by two testing personnel when testing and issuing relevant reports. They were then reviewed by a more senior physician. The cutoff values of these tumor makers were previously established by our laboratory by taking into account several environmental factors (among which diet, living conditions, and patient selection).^20^ Thus, cutoff values were set as follows: CEA <3 μg/L, CYFRA21‐1 < 4 ng/mL, CA125 < 30 U/mL, and LDH <248 U/L. The grade of TMs was marked 0 if the LDH, CEA, CYFRA211, and CA125 levels were all in the normal range. Grade 1 if one or two of the four markers were above the normal range. Grade 2 if three or four markers were above the normal range.

PET/CT was carried out with a Discovery STE PET/CT system (GE Medical Systems, USA). 18F‐FDG was provided by the PET/CT center of Shanxi Cancer Hospital with radiochemical purity of more than 95%. Fasting blood glucose amounts were less than 11 mmol/L prior to scanning. Scanning was conducted 60 min upon intravenous administration of 18F‐FDG (0.12–0.15 mCi/kg). PET employed the 3‐dimensional mode with the following parameters: 3.75 mm/slice. CT was carried out at 120 kV, 200 mA, 0.8 s/lap, and 22.5 mm/s bed speed. After that, imaging was immediately performed from skull top to upper femur (6–8 bed position and 3 min for each bed position). PET scans underwent reconstruction and attenuation based on CT images. Image illustration utilized the Xeleris Workstation (GE Medical Systems).

The image data were retained by the imaging department and the nuclear medicine department. The PET/CT data were examined by two nuclear medicine radiologists with more than 5 years of experience in an independent fashion to a consensus. Maximum tumor diameters were measured and regions of interest (ROIs) were drawn, and the SUVmax and the primary tumor size were automatically calculated by the computer.

### 2.4 Feature selection and model development and validation

Univariate analysis and multivariate logistic regression analysis were performed to search for meaningful clinical parameters. pSUVmax, LDH, CEA, CYFRA21‐1, CA125, grade of TMs, and a combined model (imaging + clinical) were developed for predicting distant metastasis. To evaluate the predictive power of each model, the receiver operating characteristic (ROC) curve analysis was constructed and areas under the curves (AUCs), sensitivity, specificity, positive predictive value, and negative predictive value were calculated.^21^, ^22^

### 2.5 Statistical analysis

All statistical analysis was performed by SPSS 22.0 (SPSS, USA). Continuous variates conforming to normal distribution are mean ± standard deviation (SD) and were compared by Student's t test. Continuous variates skewedly distributed were presented as median (interquartile range, IQR) and compared by the Wilcoxon test. Categorical variables were reported as frequency and compared by the chi‐square test. Variates with *p* < 0.05 in univariate analysis were included to binary logistic regression analysis to identify risk factors for distant metastasis of lung adenocarcinoma. Two‐sided *p* < 0.05 was deemed to indicate statistical significance.

## 3 RESULTS

**FIGURE 1:**
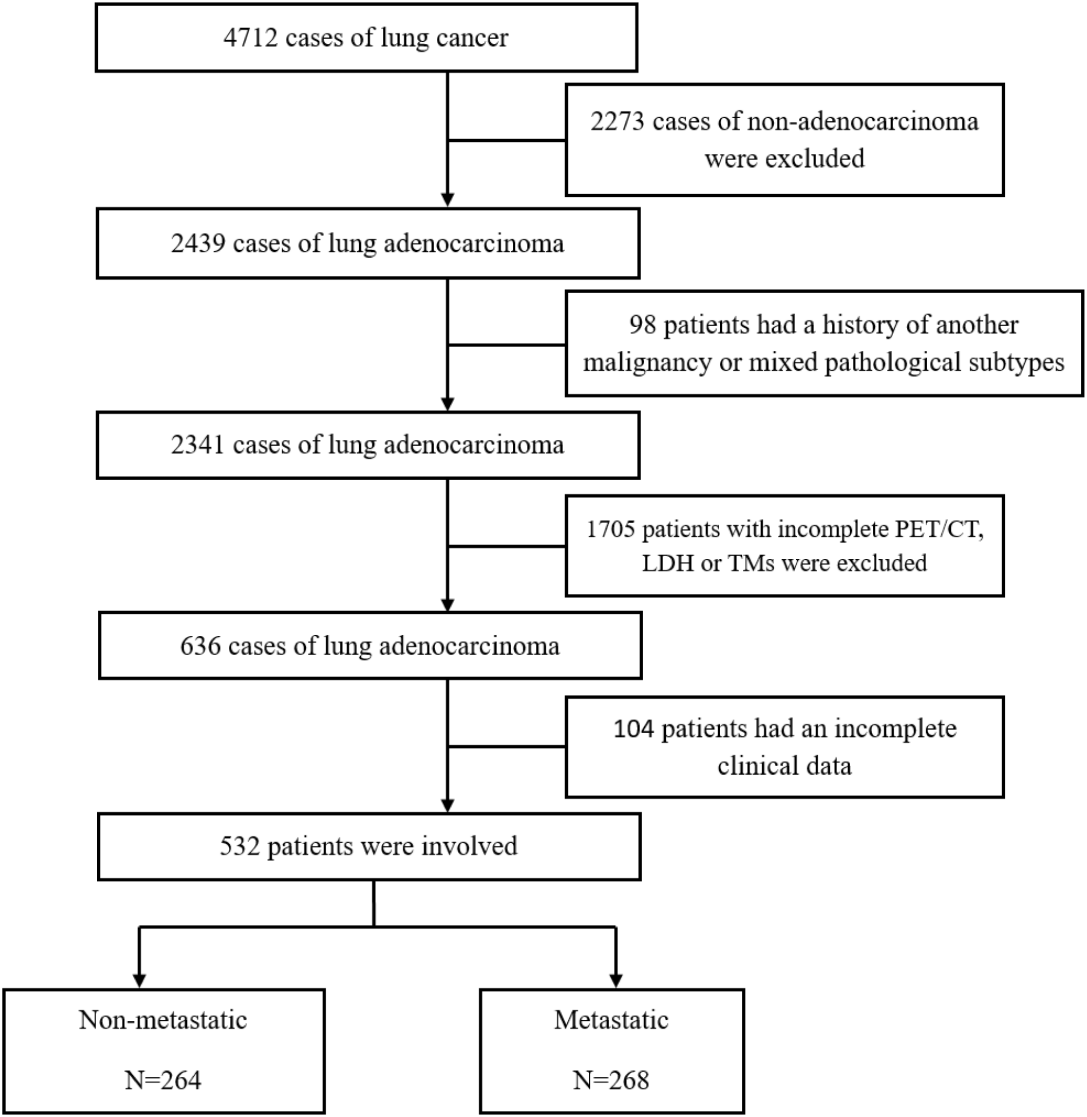
Study flowchart.

Totally 4712 cases of lung cancer were enrolled, including 2273 cases with non‐adenocarcinoma, 98 patients with previous cancer or multiple pathological subtypes, 1705 patients with incomplete PET/CT, LDH, or TMs and 104 patients with an incomplete clinical data. Finally, 532 de novo lung adenocarcinomas with available PET/CT and TMs were selected (Figure 1). Mean patient age was 60.8 ± 9.7 years (30 to 88 years); 268 (50.38%) cases were metastatic, and 224 (42.1%) were women. Patients with distant metastasis contained 136 (50.70%) and 132 (49.30%) cases with one and many metastatic sites, respectively. Additionally, the commonest metastatic sites included brain, liver, and bone, accounting for 44.7%, 26.9%, and 20.1%, respectively. The tumor statement and nodal involvement were significant difference between metastatic and non‐metastatic cases (both *p* < 0.001). pSUVmax was remarkably elevated in metastatic cases compared with non‐metastatic cases [11.17 (6.18) vs. 7.32 (5.50), *p* < 0.001] and serum TMs such as LDH [212.00 (86.00) vs. 194.50 (58.50), *p* < 0.001], CEA [8.50 (35.29) vs. 2.61 (5.55), *p* < 0.001], CYFRA21‐1 [3.58 (10.69) vs. 1.50 (2.31), *p* < 0.001], and CA125 [25.30 (66.47) vs. 5.13 (11.97), *p* < 0.001] were all elevated in the metastatic group compared with the non‐metastatic group (Table 1).

**TABLE 1:** Characteristics of patients with lung adenocarcinoma.

Characteristics	Non‐metastatic (*N* = 264)	Metastatic (*N* = 268)	*p*
Age (years), mean ± SD	61.4 ± 9.3	60.1 ± 10.0	0.119
Gender (*n*, %)			0.305
Male	147 (55.7)	161 (60.1)	
Female	117 (44.3)	107 (39.9)	
Smoking status, *n* (%)			0.914
Never	148 (56.1)	149 (55.6)	
Ever	116 (43.9)	119 (44.4)	
Tumor stage, *n* (%)			<0.001
1	100 (37.9)	36 (13.4)	
2	88 (33.3)	110 (41.0)	
3	53 (20.1)	43 (16.0)	
4	23 (8.7)	79 (29.5)	
Nodal involvement, *n* (%)			<0.001
0	123 (46.6)	28 (10.4)	
1	47 (17.8)	29 (10.8)	
2	58 (22.0)	107 (39.9)	
3	36 (13.6)	104 (38.8)	
TTF‐1, *n* (%)			0.070
Negative	22 (13.3)	29 (21.2)	
Positive	143 (86.7)	108 (78.8)	
Naspin A, *n* (%)			0.370
Negative	43 (27.9)	29 (23.2)	
Positive	111 (72.1)	96 (76.8)	
CK 7, *n* (%)			0.143
Negative	2 (1.9)	6 (7.1)	
Positive	102 (98.1)	79 (92.9)	
pSUVmax	7.32 (5.00, 10.50)	11.17 (8.22, 14.40)	<0.001
Primary tumor size (mm)	2.85 (2.03, 3.97)	3.00 (2.10,4.30)	0.222
Tumor markers			
LDH (U/L)	194.50 (167.5, 226)	212.00 (177.00, 263.00)	<0.001
CEA (μg/L)	2.61 (1.00, 6.55)	8.50 (2.15, 37.44)	<0.001
CYFRA21‐1 (ng/mL)	1.50 (0.66, 2.97)	3.58 (1.49, 12.18)	<0.001
CA125 (U/mL)	5.13 (2.19, 14.16)	25.30 (8.19, 74.66)	<0.001
Grade of TMs			<0.001
0	116 (43.9)	20 (7.5)	
1	134 (50.8)	165 (61.6)	
2	14 (5.3)	83 (31.0)	

Abbreviations: CA125, carbohydrate antigen 125; CEA, carcinoembryonic antigen; CYFRA 21‐1, cytokeratin‐19 fragment; IQR, interquartile range; LDH, lactate dehydrogenase; pSUVmax, SUVmax of the primary lesion; TMs, tumor markers.

Multivariable logistic regression analysis showed that pSUVmax (OR = 1.136, 95%CI 1.080–1.196, *p* < 0.001), tumor statement (OR = 1.331, 95%CI 1.075–1.648, *p* = 0.009), nodal involvement (OR = 1.590, 95%CI 1.294–1.952, *p* < 0.001), CEA (OR = 1.009, 95%CI 1.002–1.016, *p* = 0.017), and grade of TMs (OR = 2.883, 95%CI 1.884–4.411, *p* < 0.001) were independently associated with distant metastasis of de novo lung adenocarcinoma (Table 2).

**TABLE 2:** Multivariable logistic regression analyses of lung adenocarcinoma metastasis.

	OR (95%CI)	*p*
pSUVmax	1.136 (1.080–1.196)	<0.001
Tumor statement	1.331 (1.075–1.648)	0.009
Nodal involvement	1.590 (1.294–1.952)	<0.001
LDH	1.001 (0.999–1.003)	0.312
CA125	1.000 (0.997–1.002)	0.804
CYFRA21‐1	1.007 (0.988–1.027)	0.473
CEA	1.009 (1.002–1.016)	0.017
Grade of TMs	2.883 (1.884–4.411)	<0.001
0	Reference	
1	3.397 (1.909–6.045)	
2	8.719 (3.648–20.839)	

Abbreviations: CA125, carbohydrate antigen 125; CEA, carcinoembryonic antigen; CYFRA 21‐1, cytokeratin‐19 fragment; LDH, lactate dehydrogenase; pSUVmax, SUVmax of the primary lesion.

**FIGURE 2:**
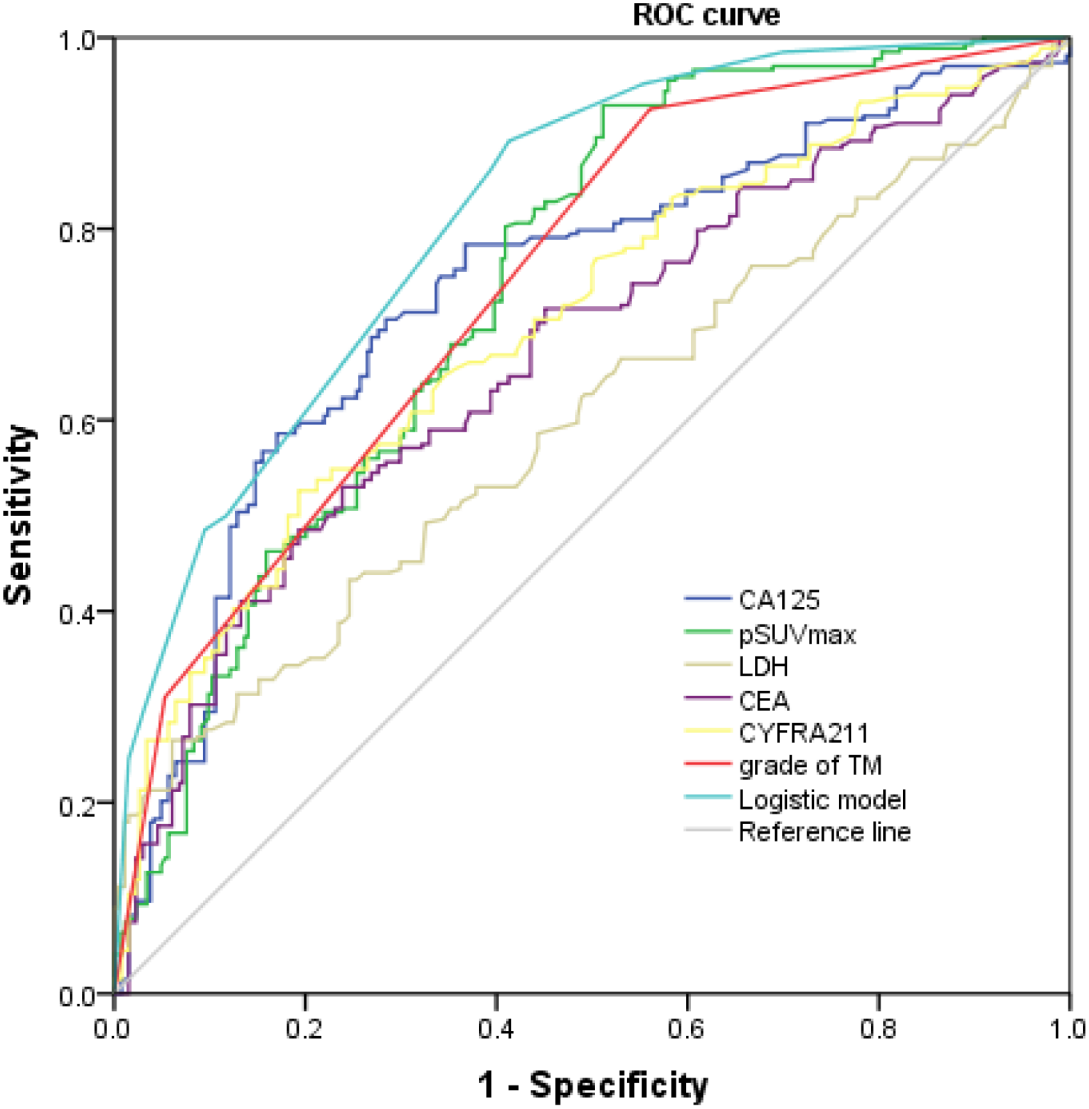
Receiver operating characteristic (ROC) curve analysis of different markers to predict the metastatic status.

The AUC values of pSUVmax, LDH, CEA, CYFRA21‐1, CA125, and grade of TMs for predicting distant metastasis were 0.742, 0.601, 0.671, 0.700, 0.736, and 0.745, respectively. Furthermore, the combination of pSUVmax, LDH, CEA, CYFRA21‐1, CA125, and grade of TMs in predicting distant metastasis had an AUC value of 0.816 (95%CI 0.781–0.851), with sensitivity of 89.2%, specificity of 58.7%, positive predictive value of 73.7%, and negative predictive value of 79.7%, respectively (Table 3 and Figure 2).

**TABLE 3:** ROC curve analysis of different indicators.

Indicators	AUC	95%CI	*p*	Cutoff value	Sensitivity	Specificity	Positive predictive value	Negative predictive value	Kappa
pSUVmax	0.742	0.700–0.784	<0.001	7.195	92.9	48.9	64.3	83.8	69.9
LDH	0.601	0.553–0.649	<0.001	260	26.5	93.9	81.6	55.7	60.0
CEA	0.671	0.626–0.717	<0.001	9.08	48.5	80.7	71.8	60.7	64.5
CYFRA21‐1	0.700	0.656–0.744	<0.001	3.28	52.6	80.7	73.4	62.6	66.5
CA125	0.736	0.693–0.779	<0.001	9.51	70.5	71.6	71.5	70.3	70.9
Grade of TMs	0.745	0.703–0.786	<0.001		92.5	43.9	62.6	85.3	68.4
Combination	0.816	0.781–0.851	<0.001	‐	89.2	58.7	73.7	79.7	76.3

*Note*: Combination included pSUVmax, LDH, CEA, 211, CA125, and the grade of TMs.

Abbreviations: AUC, area under the curve; CA125, carbohydrate antigen 125; CEA, carcinoembryonic antigen; CI, confidence interval; CYFRA 21‐1, cytokeratin‐19 fragment; LDH, lactate dehydrogenase; pSUVmax, SUVmax of the primary lesion; ROC, receiver operating characteristic.

## 4 DISCUSSION

This study found that pSUVmax combined with serum TMs level may predict distant metastasis of de novo lung adenocarcinoma. The results may help clinicians better identify distant metastasis of newly diagnosed lung adenocarcinoma.

The detection of distant metastasis is one of the most important ingredient factors for staging, individualized systemic therapeutic strategies, and prognosis evaluation^23^ However, there is still a lack of accurate diagnosis methods for distant metastasis of de novo lung adenocarcinoma. Studies have shown that both imaging and TMs can be applied in diagnostic approaches for distant metastasis of lung adenocarcinoma, but the prediction accuracy of a single indicator is insufficient.^14^, ^18^, ^19^, ^24^ Recently, the joint application of ^18^F‐FDG PET/CT and TMs in the diagnosis of recurrence and metastasis has attracted huge attentions in lung cancers.^9^, ^25^ 18F‐FDG PET/CT can provide anatomical and metabolic information about lesions.^24^ In the present study, compared with the non‐metastatic patients, the pSUVmax of the primary lesion was significantly elevated in the metastatic patients. ROC curves have been shown to be a useful tool in assessing the ability of a model to predict a wide range of outcomes from overall survival in multi‐organ cancer^21^ and identifying SNARE proteins with computational frameworks.^22^ Here, we use ROC curve analysis and found the AUC of pSUVmax to diagnose distant metastasis was 0.742. This is the first study to assess pSUVmax for its diagnostic value in distant metastasis of lung adenocarcinoma. Previous studies mainly focused on the relationship between pSUVmax and lymph node metastasis.^9^, ^26^ Therefore, pSUVmax lesions are important diagnostic indicators for both distant metastasis and lymph node metastasis of NSCLC.

LDH and TMs are important indicators in the diagnosis, treatment decision, and prognosis of lung cancer as well.^18^, ^27^ LDH is a crucial enzyme in the glycolytic process and is associated with survival and proliferation in cancer‐initiating cells.^24^ Studies have shown that elevated levels of LDH are related to metastasis and a poor prognosis,^4^, ^27^ which is consistent with this findings. The serum levels of CEA, CYFRA21‐1, and CA125 are also frequently detected in lung cancers for diagnosis, therapeutic strategies, response assessment, and prognostic approaches.^13^, ^15^, ^28^, ^29^ The current results corroborated the above studies. pSUVmax, and the serum LDH, CEA, CYFRA211, and CA125 amounts assessed in this work were increased and prone to change in metastatic patients compared with non‐metastatic patients. The evaluation of such parameters may be helpful in clinic for assessing whether lung adenocarcinomas have elevated odds of developing distant metastasis at presentation. Previously, researchers had paid more attention to differentiate between lymph node metastasis and benign lymph node lesions.^9^, ^25^ Nevertheless, it is equally important to identify the presence of distant metastasis for proper patient management.^30^ This work highly indicates proper use of pSUVmax, LDH, CEA, CYFRA211, and CA125 could indicate distant metastasis. In future it may be possible to design a nomogram based on this model to use this information to evaluate the risk of distant metastasis in patients with lung adenocarcinoma.

This study also had multiple limitations. First, it had a single‐center retrospective design, which limits data analysis to a certain extent and may have introduced some bias into the study. Secondly, due to incomplete follow‐up information, a predictive model for survival was not established. Third, if there were any errors in the original diagnosis of distant metastasis in this study that could be a potential weakness, because the diagnosis was used to compare the pSUVmax, LDH, CEA, CYFRA211, and CA125 results. The original diagnosis of metastases was based on CT, ultrasound, MRI, PET scan, emission CT, fine‐needle aspiration biopsy, pleural effusion cytology, and/or biopsy. More than two pathologists and physicians with more than 5 years of work experience used this information alongside the eighth edition of the TNM staging system for lung cancer. It was important to base the diagnosis in clinic on various different approaches because there may be variations in reliability between methods. However, if both pathologists were considered to be distant metastasis, it was considered to be distant metastasis. If both pathologists do not match, the case was eliminated from the study. Fourth, the single‐center nature of the study means that while these results may be typical of patients at a provincial hospital in Shanxi Province, they are not necessarily representative of patient populations in other regions. Fifth, some patients were excluded from the study with missing data, which might be another source of bias. Finally, because the patients in this study attended the hospital over a period of nearly 5 years, while there were no major changes in diagnostic or treatment processes in the hospital at that time, there may have been some slight differences over such a period of time, which may influence the results.

## 5 CONCLUSIONS

Overall, pSUVmax combined with TMs may have predictive value for distant metastasis of de novo lung adenocarcinomas. Large multicenter studies are required to confirm these data in the future.

## AUTHOR CONTRIBUTIONS

**Baoli Jin:** Conceptualization (equal); formal analysis (equal); writing – original draft (equal); writing – review and editing (equal). **Xiaolian Wen:** Data curation (equal); investigation (equal); writing – original draft (equal); writing – review and editing (equal). **Hanji Tian:** Formal analysis (equal); methodology (equal); writing – original draft (equal); writing – review and editing (equal). **Hongxia Guo:** Formal analysis (equal); methodology (equal); writing – original draft (equal); writing – review and editing (equal). **Mingyan Hao:** Supervision (equal); writing – original draft (equal); writing – review and editing (equal). **Jing Wu:** Formal analysis (equal); writing – original draft (equal); writing – review and editing (equal). **Xiaomin Li:** Methodology (equal); writing – original draft (equal); writing – review and editing (equal). **Yuejun Ren:** Methodology (equal); writing – original draft (equal); writing – review and editing (equal). **Xin Wang:** Investigation (equal); methodology (equal); writing – original draft (equal); writing – review and editing (equal). **Xiaolu Ren:** Conceptualization (equal); formal analysis (equal); writing – original draft (equal); writing – review and editing (equal).

## FUNDING INFORMATION

The current project was funded by the Fund program for the scientific activities of selected returned overseas professionals in Shanxi province (No. 2021005), the Shanxi Province basic applied research project (No. 202103021223447, 20210302124611 and 202203021212066), and Youth Foundation of Health Commission of Shanxi Province (No. 2020058).

## CONFLICT OF INTEREST STATEMENT

The authors declare no competing interests.

## ETHICS STATEMENT

This study had approval from the Institutional Review Board of Shanxi Cancer Hospital (2022007), who waived the requirement for informed consent because of its retrospective nature. The study protocol followed the 1964 Declaration of Helsinki and its later amendments.

## Data Availability

All data generated or analyzed during the present study are included in this published article.
